# Electroacupuncture Attenuates Peripheral Neuropathy Development in Hyperglycemic Rats and Is Associated With NLRP3 Inflammasome Modulation

**DOI:** 10.1155/jdr/6233881

**Published:** 2026-10-19

**Authors:** Ziyue Zhu, Qinglan Zou, Chengguo Su, Xi You, Weijian Zeng, Nanxi Li, Bumrungtham Thitinun, Ying Li, Junpeng Yao, Wei Zhang

**Affiliations:** ^1^ School of Acupuncture-Moxibustion Chengdu University of Traditional Chinese Medicine Chengdu Sichuan China cdutcm.edu.cn; ^2^ School of Nursing Chengdu University of Traditional Chinese Medicine Chengdu Sichuan China cdutcm.edu.cn; ^3^ Graduate School Chengdu University of Traditional Chinese Medicine Chengdu Sichuan China cdutcm.edu.cn; ^4^ Key Laboratory of Acupuncture for Senile Disease (Chengdu University of TCM) Ministry of Education Chengdu Sichuan China meb.gov.tr

**Keywords:** diabetic peripheral neuropathy, electroacupuncture, inflammation

## Abstract

**Background:**

This study employed a high‐energy diet and streptozotocin (STZ) injection to establish a rat model of diabetic peripheral neuropathy (DPN), focusing on inflammatory responses. The aim was to investigate the mechanism by which electroacupuncture (EA) improves the neuroinflammatory microenvironment in diabetic rats and attenuates DPN development.

**Method:**

Twenty‐four male SD rats were randomly assigned using a random number table into control, model, and EA groups, with eight rats in each group. A DPN model was established using a high‐energy diet combined with STZ injection. The EA group received intervention immediately after successful diabetic modeling, whereas intervention ceased once DPN modeling was established in the model group. During the intervention period, random blood glucose levels and paw withdrawal latency (PWL) were recorded weekly for rats in each group. After intervention, the neurophysiological status of rats was assessed. The ultrastructure of Schwann cells in the sciatic nerve was observed using transmission electron microscopy. Western Blot and RT‐PCR were employed to detect the protein levels and mRNA expression of ASC, caspase‐1, NLRP3, and P62. ELISA was implemented to measure IL‐18 and IL‐1*β* levels.

**Results:**

Compared with the control, model rats exhibited significantly elevated random blood glucose and PWL levels (*p* < 0.0001, *p* < 0.0001), markedly reduced motor nerve conduction velocity (MNCV) (*p* < 0.0001), and pronounced damage to Schwann cell structure. ASC, caspase‐1, NLRP3, and P62 levels were markedly elevated (*p* < 0.01, *p* < 0.0001, *p* < 0.0001, *p* < 0.001; *p* < 0.001, *p* < 0.01, *p* < 0.05, *p* < 0.001), whereas IL‐18 and IL‐1*β* levels were notably elevated (*p* < 0.0001, *p* < 0.0001). Compared with the model group, rats in the EA group displayed no marked difference in random blood glucose (*p* > 0.05), but reduced PWL (*p* < 0.0001), increased MNCV (*p* < 0.0001), and milder structural damage to Schwann cells. ASC, caspase‐1, NLRP3, and P62 levels were considerably reduced (*p* < 0.01, *p* < 0.0001, *p* < 0.001, *p* < 0.001; *p* < 0.05, *p* < 0.05, *p* < 0.05, *p* < 0.05), whereas IL‐18 and IL‐1*β* levels were notably diminished (*p* < 0.001, *p* < 0.0001).

**Conclusion:**

EA may protect peripheral nerve tissue morphology and function by suppressing NLRP3 inflammasomes, thereby alleviating inflammatory responses and potentially attenuating the onset and progression of DPN. This process may be associated with autophagy; but further mechanistic studies are needed to establish causality.

## 1. Introduction

Diabetes is a globally prevalent chronic disease, with 578 million people projected to have the condition by 2030 and 700 million by 2045 [1]. As a common complication, diabetic peripheral neuropathy (DPN) affects approximately 50% of people with diabetes, making it a significant health issue [2, 3]. Although hypoglycemic agents, analgesics, microcirculation enhancers, and neurotrophic agents are available [2], DPN remains incurable because of its complex pathogenesis and insidious onset, with symptoms often irreversible once manifested [4]. Therefore, attenuating DPN development is of paramount importance [3, 5], prompting growing interest in nonpharmacological strategies such as acupuncture and specific exercise regimens [6, 7]. Acupuncture, as a traditional nonpharmacological therapy, exerts neuroprotective effects on peripheral nerves by improving nerve conduction function, promoting myelin reconstruction, and facilitating axonal regeneration [8–10]. It has been applied to prevent chemotherapy‐induced peripheral neuropathy [11]. Recent research further suggests that early acupuncture intervention may prevent DPN‐related pain [12], though its mechanisms require further investigation.

Inflammation is pivotal in DPN development. Prolonged hyperglycemia activates the innate immune system, inducing chronic inflammation that damages nerves by disrupting mitochondrial function and vascular structure [13–15]. Among these mechanisms, the assembly and activation of the NOD‐like receptor pyrin domain‐containing 3 (NLRP3) inflammasome, a central component of the inflammatory response, has been demonstrated as a major factor driving DPN pathogenesis [16]. It can be activated by excessive reactive oxygen species, subsequently inducing the activation of inflammatory cytokines IL‐18 and IL‐1*β*, thereby causing excessive inflammatory responses and triggering DPN [17]. Inhibiting this inflammatory process can significantly alleviate DPN symptoms [18].

Schwann cells, the principal glial cells responsible for myelination and axonal support in peripheral nerves, are particularly vulnerable to hyperglycemia‐induced NLRP3 inflammasome activation. Upon activation, NLRP3 recruits ASC and procaspase‐1 to form a functional complex, which cleaves procaspase‐1 into active caspase‐1. This protease not only matures IL‐1*β* and IL‐18 but also cleaves gasdermin D, inducing pyroptosis‐a lytic and proinflammatory form of cell death [19]. Pyroptotic Schwann cells lose their ability to maintain myelin sheaths and release damage‐associated molecular patterns, which further aggravate local inflammation and directly disrupt myelin integrity. Progressive demyelination, in turn, impairs saltatory conduction along nerve fibers, leading to reduced motor nerve conduction velocity (MNCV) and sensory deficits [20]‐hallmark features of DPN. Thus, the cascade from NLRP3 activation to Schwann cell injury, demyelination, and conduction failure represents a critical pathogenic axis in DPN.

Electroacupuncture (EA) is a therapeutic modality that combines traditional acupuncture with the application of low‐intensity electrical stimulation through acupuncture needles. It delivers controlled electrical pulses to acupoints, thereby amplifying the analgesic and neuromodulatory effects of manual acupuncture [21]. The anti‐inflammatory effects of EA have been widely recognized. Research indicates that EA activates the vagus nerve‐adrenergic axis to exert systemic anti‐inflammatory effects [22] and suppresses neuroinflammation and NLRP3 inflammasomes in various disease models, thereby improving related pathological states [23–25]. Consequently, EA may attenuate the onset and progression of DPN by inhibiting NLRP3 inflammasomes and alleviating inflammatory responses. Additionally, the autophagy adapter protein P62 specifically recognizes and binds to NLRP3 inflammasomes to promote autophagic degradation [26]. Hence, this study also included it as an observational indicator to preliminarily explore whether autophagy may be involved in the regulation of NLRP3 inflammasomes by EA.

## 2. Materials and Methods

### 2.1. Experimental Animals

Twenty‐four male Sprague‐Dawley rats (8 weeks old, 200 ± 12 g) were procured from Chengdu Dashuo Biotech (Chengdu, China) and reared in SPF facilities at the Laboratory Animal Center of Chengdu University of Traditional Chinese Medicine with controlled humidity, temperature, and circadian rhythm. These rats were maintained according to an experimental protocol approved by Animal Welfare and Ethics Committee (2023‐06) of Chengdu University of Traditional Chinese Medicine. After tissue collection, euthanasia was performed by administering 3% Pentobarbital sodium (PUHUIFEN, China) at a dosage three times the anesthetic dose.

### 2.2. Induction of DPN

After adaptive feeding for 1 week, rats were randomly assigned to the control, model, and EA groups, with eight rats in each group. Except for the control group, all other groups received high‐energy feed and STZ intraperitoneal injection for DPN modeling. After 8 weeks of high‐energy diet feeding, rats fasted for 12 h and then were administered STZ (Sigma, 35 mg/kg) via intraperitoneal injection under dark conditions. During the intervention period, the model and EA groups continued high‐energy feeding to maintain the model effect. Successful diabetes modeling was defined as random blood glucose ≥ 16.7 mmol/L measured in the rat tail vein. Successful DPN modeling was defined as a notable decrease or increase in the thermal pain threshold compared with the control group.

### 2.3. EA Intervention

The acupoints selected for needling were Zusanli (ST36), Sanyinjiao (SP6), and Weiwanxiashu (EX‐B3), which are the three most commonly used acupoints for acupuncture treatment of DPN [27]. After restraining the rats and disinfecting the skin, stainless steel needles (*Φ*0.13 × 13 mm, Hwato, Suzhou Medical Supplies) were inserted into these acupoints to a depth of 1–2 mm. The needles at the Zusanli (ST36) and Sanyinjiao (SP6) points were connected to an EA device (Hwato‐SDZ‐II, Suzhou Medical Supplies). Parameters were set to sparse‐dense wave at 2 Hz/15 Hz, as research indicates these settings demonstrate superior efficacy in DPN treatment [28]. The current intensity was set at 2 mA, which induced slight tremors in the rat′s lower limbs. Treatment started after successful establishment of the diabetic model and ceased once DPN modeling was confirmed in the model group. Sessions lasted 20 min each, once daily for 5 consecutive days per course. A 2‐day rest period followed each course. This experiment comprised five treatment courses.

### 2.4. Random Blood Glucose and Paw Withdrawal Latency (PWL) Testing

Random blood glucose and PWL testing were conducted on rats in each group before intervention and after each treatment course during the intervention period; random blood glucose testing was performed at the same time of day (17:00–19:00) to control for diurnal variation. The specific methods were as follows: (1) Random blood glucose: After disinfection, the skin of the rat′s tail was rapidly punctured with a blood‐collecting needle, and a complete blood droplet was squeezed out. The sample was then tested using a blood glucose meter (Sinocare‐GA‐3, Shenzhen Sannuo Biosensor Co. Ltd., China). (2) Rats were placed on a 50°C constant‐temperature hot plate (SR15‐KW‐RB, Oriental Glass Beijing Technology Co. Ltd., China). The time at which each rat exhibited paw withdrawal or struggle was recorded as the PWL. To prevent paw burns, a cutoff time of 30 s was set.

### 2.5. Testing of MNCV

Following the intervention, MNCV testing was performed. Rats were anesthetized with intraperitoneal injection of 3% pentobarbital sodium (PUHUIFEN, China) and then fixed in a prone position. Stimulation and recording electrodes were placed at the sciatic notch (i.e., the site of sciatic nerve exit) and the site where the sciatic nerve passed through the ankle joint, respectively. These electrodes were connected to the PowerLab data acquisition and analysis system (PL3508). The time taken for action potentials to traverse the recording electrode was observed. Subsequently, the distance between the stimulating electrode and the recording electrode was measured on the body surface. Finally, MNCV (m·s^−1^) was calculated according to the formula:

MNCV=distance between stimulating and recording electrodes/conduction time.

### 2.6. Acquisition of Serum and Sciatic Nerve

Following MNCV detection, blood was obtained from the abdominal aorta and centrifuged (3000 r/min) to obtain serum for testing IL‐18 and IL‐1*β* levels. After blood collection, the sciatic nerve was rapidly dissected and divided into two portions. One portion was placed in 3% glutaraldehyde for transmission electron microscopy (TEM) observation. The other portion was stored at −80°C for subsequent Western blot and RT‐PCR analyses.

### 2.7. TEM

The ultrastructure of Schwann cells in the sciatic nerve was inspected using TEM. Sciatic nerve tissue was fixed in 3% glutaraldehyde (12 h), dehydrated, and embedded in resin. After that, ultrathin sections (60–90 mm) were prepared, dyed with lead citrate (1–2 min), and observed under a TEM.

### 2.8. Enzyme‐Linked Immunosorbent Assay (ELISA)

IL‐18 and IL‐1*β* are downstream inflammatory factors of NLRP3 complexes. They were quantified using ELISA kits (Rat IL‐18 ELISA KIT, Rat IL‐1*β* ELISA KIT, JINGMEI BIO, China). After sample addition, washing, color development, and termination solution addition, optical density (OD) values were gauged using a microplate reader.

### 2.9. Western Blot

Western blot analysis was performed to quantify the expression levels of ASC, caspase‐1, NLRP3, and P62 in the sciatic nerve. Briefly, rat sciatic nerve tissue was ground in a high‐speed, low‐temperature tissue grinder with RIPA lysis buffer (Servicebio, China) at a mass‐to‐volume ratio of 1:10 to extract total protein. Protein concentration was determined using the BCA assay kit (Beyotime, China). An equal amount of total protein was loaded and subjected to SDS‐PAGE under a constant voltage of 100 V. The separated proteins were transferred to PVDF membranes (Sigma‐Aldrich, United States) for 1–2 h. After that, the membrane was blocked with TBST solution containing 5% nonfat milk at ambient temperature for 2 h, and probed with antibodies against ASC (1:1000), caspase‐1 (1:2000), NLRP3 (1:1000), P62 (1:1000, all from Abcam, United Kingdom), and *β*‐actin (1:50000, Abclonal, China) overnight at 4°C. After TBST washing, the membrane was incubated at room ambient for 2 h with the corresponding HRP‐labeled secondary antibody (1:5000, Abclonal, China).

The membrane was developed using ECL chemiluminescent reagent, and images were captured using the Tanon fluorescence image analysis system. Finally, the integrated OD values of the target bands were analyzed using Gel‐Pro analyzer 4 software. The quantification procedure was as follows: Western blot protein bands were analyzed by densitometry using Gel‐Pro analyzer 4.0 software (Media Cybernetics, United States). Automatic edge detection was performed, and the selection was then manually adjusted to include the entire target band as fully as possible in order to minimize error. Results were output as integrated optical density (IOD) values. All target protein expression levels were normalized to *β*‐actin as the internal loading control. Data are presented as the ratio of target protein IOD to *β*‐actin IOD. Band intensities were quantified using Gel‐Pro analyzer 4.0 software, and *β*‐actin served as the internal loading control. The coefficient of variation (CV) of *β*‐actin band intensities across all lanes was maintained below 15%.

### 2.10. RT‐PCR

RT‐PCR was used to determine mRNA expression levels of ASC, caspase‐1, NLRP3, and P62 in the sciatic nerve. Specifically, total RNA was extracted from sciatic nerve tissue. Its purity was assessed, and it was then reverse transcribed into cDNA for PCR amplification. The amplification efficiency of all primers was validated using the standard curve method, with efficiencies falling between 90% and 110%. The specificity of PCR products was confirmed by melting curve analysis, and a single melting peak was observed in all reactions. Relative gene expression was calculated using the 2^−*ΔΔ*CT^ method with *β*‐actin as the reference gene. All samples for each target gene were amplified on a single 96‐well plate, thus eliminating inter‐run variation. Table 1 outlines primer sequences.

**Table 1:** Primer sequences of ASC, caspase‐1, NLRP3, and P62.

Primer	Forward	Reverse	Product length	Tm value
*β*‐actin	gggaaatcgtgcgtgacatt	gcggcagtggccatctc	76 bp	56/60.8
ASC	atggaagagtctggagctgtgg	aatgagtgcttgcctgtgttgg	99 bp	59.2/58.5
Caspase‐1	ttgccctttagaaatagcccagaag	tcaacatcagctccgactctcc	150 bp	56.9/59
NLRP3	agacctccaagaccacgactg	ccatccgcagccaatgaacag	127 bp	59.2/59.7
P62	gcctatggacaagatgactggaatg	tgctctctgtatgctcccttcac	86 bp	57.6/58.9

*Note:* Product length; Tm value; primer source: The full gene sequences were retrieved from the National Center for Biotechnology Information (NCBI) database. Primer Premier software was used to design and screen gene‐specific primers. All primers were synthesized and ULTRAPAGE‐purified by Sangon Biotech (Shanghai) Co. Ltd.

### 2.11. Statistical Analysis

Data were statistically analyzed and graphed using GraphPad Prism 10.4.1. One‐way analysis of variance (ANOVA) was performed for pairwise comparisons. For groups with homogeneous variance, pairwise comparisons were conducted using Tukey′s test; for groups with heterogeneous variance, Brown–Forsyth′s test was applied. For data not meeting normality assumptions, the Kruskal–Wallis rank‐sum test was employed.

Sample size was determined by an a priori power analysis using G∗Power 3.1.9.7. Based on previously reported effects of EA on PWL in diabetic neuropathy rat models [29], where large effect sizes (Cohen′s f) ranging from 0.60 to over 1.0 have been observed, we conservatively assumed a large effect size of *f* = 0.60 for the primary outcome. With *α* = 0.05, power (1 − *β*) = 0.80, and three groups, the required total sample size was calculated as 21. Allowing for a model failure or mortality rate of approximately 10%–15%, we enrolled eight rats per group (total *N* = 24).

## 3. Results

### 3.1. EA Protects Sciatic Nerve Function and Morphology and Attenuates DPN Development

Nerve fiber damage is a key characteristic in DPN development. Small nerve fiber injury often manifests as loss or hypersensitivity of temperature sensation, whereas large nerve fiber injury is characterized by decreased MNCV. As shown in Figure 1B, PWL values were comparable across groups before intervention (*p* > 0.05). After 5 weeks, the model group exhibited greatly elevated PWL (vs. control group) (*p* < 0.0001), indicating small nerve fiber injury. In contrast, the EA group demonstrated no marked difference in PWL with the control group (*p* > 0.05). Neurophysiological testing revealed (Figure 1C) that after 5 weeks of intervention, model rats exhibited markedly reduced MNCV compared with the control (*p* < 0.0001), indicating impaired large nerve fiber function. The EA group demonstrated a higher MNCV than the model group (*p* < 0.0001), whereas no marked difference was revealed between the control and EA groups (*p* > 0.05). This suggests that EA exerts potent neuroprotective effects, potentially attenuating DPN development.

**Figure 1:**
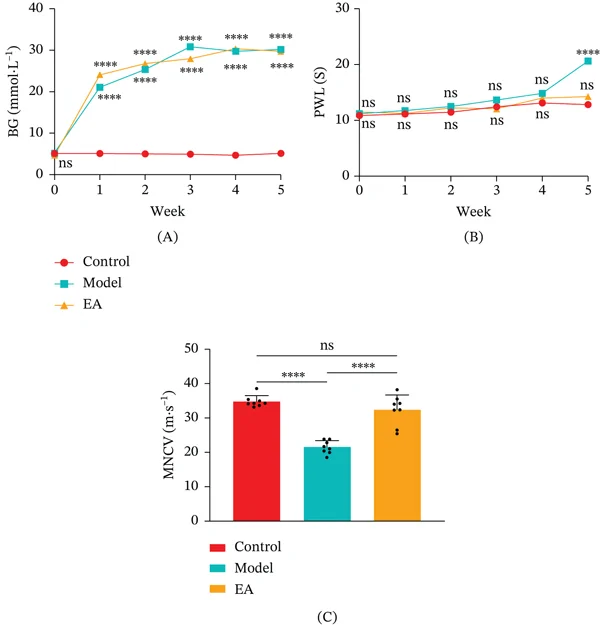
(A) Random blood glucose levels before and during intervention, *n* = 8, ^ns^ *p* > 0.05; versus the control group,  ^∗∗∗∗^ *p* < 0.0001. (B) PWL before and during intervention, *n* = 8; versus the control group, ^ns^ *p* > 0.05,  ^∗∗∗∗^ *p* < 0.0001. (C) MNCV after 5 weeks of intervention, *n* = 8; ^ns^ *p* > 0.05,  ^∗∗∗∗^ *p* < 0.0001.

TEM observations revealed that Schwann cells in the control and EA groups exhibited relatively normal morphology and intact cellular structures, whereas the model group demonstrated marked demyelination, mild mitochondrial swelling, and cystic dilation of the endoplasmic reticulum (Figure 2). This further confirms that EA possesses protective effects on peripheral nerves and may attenuate DPN.

**Figure 2:**
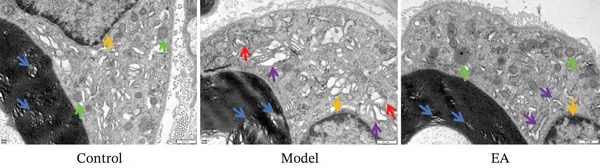
TEM (25,000× magnification). Blue arrow: demyelination; yellow arrow: cell nucleus; green arrow: mitochondria; red arrow: mild mitochondrial swelling; purple arrow: cystic dilation of rough endoplasmic reticulum. Scale bar = 500 nm.

However, EA did not exhibit a significant hypoglycemic effect. During the intervention period, random blood glucose levels in the model and EA groups were substantially raised (*p* < 0.0001, *p* < 0.0001) (Figure 1A). This observation suggests that the neuroprotective effects of EA may be less dependent on glycemic control, pointing toward glucose‐independent mechanisms. (Effect sizes and 95% confidence intervals for all nerve function outcomes are summarized in Table S1 in File S1. The original data are provided in File S2.)

### 3.2. EA Mitigates Inflammatory Responses and Protects Neural Function by Inhibiting NLRP3 Inflammasomes

NLRP3 inflammasomes and the subsequent excessive production of downstream inflammatory cytokines IL‐18 and IL‐1*β* constitute a key mechanism in DPN pathogenesis. The model group displayed remarkably elevated protein and mRNA levels of ASC, caspase‐1, and NLRP3 (vs. control group) (*p* < 0.01, *p* < 0.0001, *p* < 0.0001; *p* < 0.001, *p* < 0.01, *p* < 0.05). Following EA intervention, these levels were considerably downregulated (*p* < 0.01, *p* < 0.0001, *p* < 0.001; *p* < 0.05, *p* < 0.05, *p* < 0.05) (Figure 3A,B). ELISA results showed (Figure 4) greatly increased IL‐18 and IL‐1*β* levels in the model group vs. control group) (*p* < 0.001, *p* < 0.0001). There was no considerable difference between the EA and control groups (*p* > 0.05). In contrast, the EA group exhibited greatly lowered levels of IL‐18 and IL‐1*β* (vs. model group) (*p* < 0.01, *p* < 0.0001). This suggests that EA significantly curbs NLRP3 inflammasomes and downstream inflammatory mediators, thereby exerting a protective effect on peripheral nerves.

**Figure 3:**
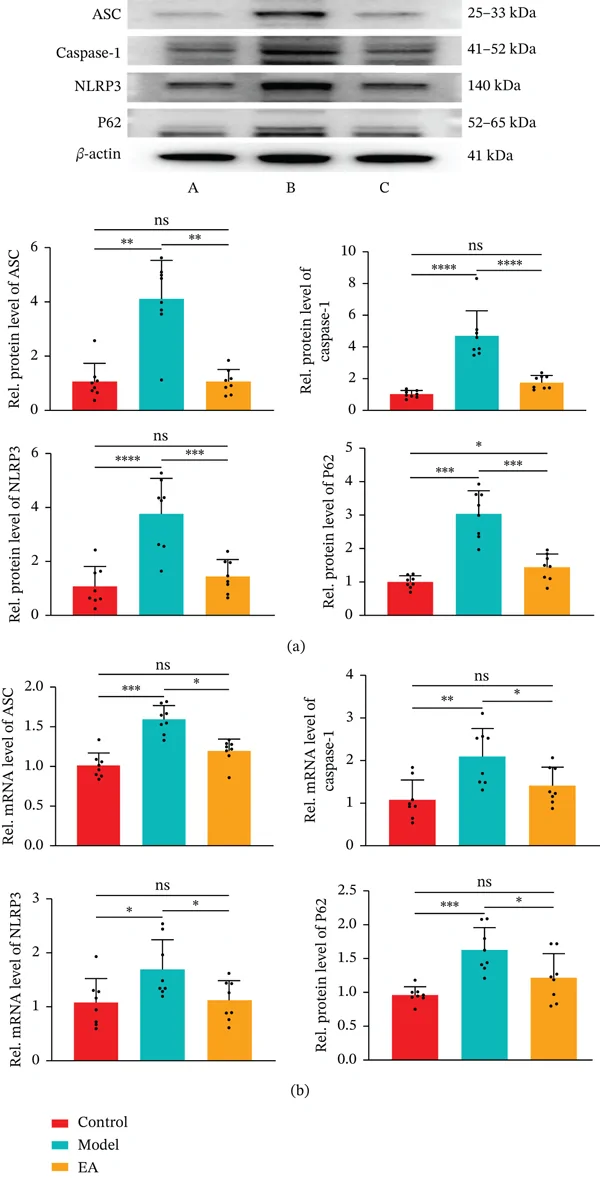
(a) Relative protein levels of ASC, caspase‐1, NLRP3, and P62; A: control group, B: model group, and C: EA group. ^ns^ *p* > 0.05,  ^∗^ *p* < 0.05,  ^∗∗^ *p* < 0.01,  ^∗∗∗^ *p* < 0.001,  ^∗∗∗∗^ *p* < 0.0001. (b) Relative mRNA levels of ASC, caspase‐1, NLRP3, and P62. ^ns^ *p* > 0.05,  ^∗^ *p* < 0.05,  ^∗∗^ *p* < 0.01,  ^∗∗∗^ *p* < 0.001.

**Figure 4:**
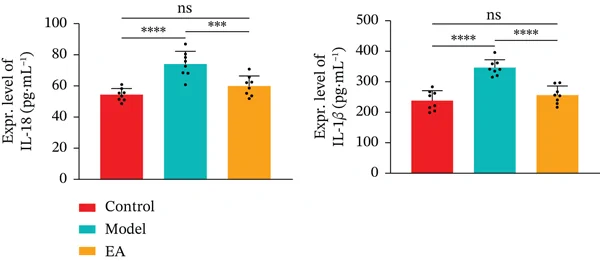
IL‐18 and IL‐1*β* levels. ^ns^ *p* > 0.05,  ^∗∗∗^ *p* < 0.001,  ^∗∗∗∗^ *p* < 0.0001.

Furthermore, since our findings indicated that EA did not exert a blood glucose‐regulating effect, this suggests its anti‐inflammatory action may not solely depend on glycemic control. Therefore, we further examined P62, an autophagy adapter protein closely associated with NLRP3 inflammasome degradation, to preliminarily explore the underlying mechanisms of EA′s anti‐inflammatory effects. Results showed substantially elevated P62 protein and mRNA levels in the model group (vs. control group) (*p* < 0.001, *p* < 0.001) but considerably reduced P62 levels in the EA group (vs. model group) (*p* < 0.001, *p* < 0.05). The corresponding effect sizes and confidence intervals for molecular markers are presented in Table S1 in File S1. The RT‐PCR data analysis and results are presented in Tables S2–S5 in File S1, the amplification curves and melt curves are shown in Figures S1–S10 in File S1, and the Western blot bands are provided in Figures S11–S15 in File S1.

## 4. Discussion

Our findings demonstrate that EA preserved peripheral nerve function and morphology in diabetic rats, as evidenced by improved PWL and MNCV, reduced structural damage to Schwann cells, and decreased expression of NLRP3 inflammasome components and downstream inflammatory cytokines. These results collectively indicate an association between EA‐mediated neuroprotection and suppression of NLRP3 inflammasome‐driven neuroinflammation. However, since we did not employ pharmacological inhibitors, activators, or genetic manipulation of NLRP3, the observed relationship should be interpreted as correlative rather than causal.

To contextualize these findings, it is helpful to first outline the pathogenic cascade linking hyperglycemia to peripheral nerve dysfunction, against which EA′s effects can be assessed. Prolonged hyperglycemia activates the NLRP3 inflammasome in Schwann cells, triggering pyroptotic cell death and demyelination, which in turn disrupts saltatory conduction and leads to sensory and motor deficits—a sequence that our experimental evidence addresses at multiple levels.

Assessing peripheral nerve function is essential to determine whether interventions can effectively protect peripheral nerves under pathological conditions. PWL and nerve conduction are representative assessment tests [30] that play a crucial role in identifying DPN. As thermal sensation is conveyed by small A*δ* and C fibers, PWL sensitively reflects the functional integrity of small nerve fibers in animal models. As one of the longest peripheral nerves, the sciatic nerve is often the first to be affected upon peripheral nerve injury. Monitoring its conduction velocity allows for timely and objective detection of pathological changes in the myelin sheath and axons of large nerve fibers [31]. Therefore, we used both approaches to evaluate the status of large and small nerve fibers and to investigate the effects of acupuncture on peripheral nerve function. Our results demonstrated that EA significantly preserved nerve fiber function in diabetic rats, thereby attenuating DPN development. At the structural level, TEM observation of Schwann cells in the sciatic nerve revealed that the ultrastructure in the EA group remained more intact. This objectively corroborates the protective effects of EA on peripheral nerves, consistent with previous findings [32, 33].

Increasing evidence confirms that neuroinflammation plays a critical role in the pathogenesis of diabetic neuropathy [34, 35]. At the core of the inflammatory response lies the initiation and activation of inflammasomes, large cytoplasmic protein complexes found in macrophages and both immune and nonimmune cells [36]. Among these, the NLRP3 inflammasome, highly expressed in glial cells and neurons, has garnered particular attention as a classical proneuroinflammatory multiprotein complex [14]. Under specific conditions, NLRP3 can bind to ASC and recruit procaspase‐1 to form complete NLRP3 inflammasomes. This complex subsequently cleaves procaspase‐1 into active caspase‐1, driving downstream inflammatory responses [37].

In the peripheral nervous system, the detrimental effects of NLRP3 activation are prominently manifested in Schwann cells [37]. Activated caspase‐1 exerts dual pathologic actions: It processes pro–IL‐1*β* and pro–IL‐18 into their mature secretory forms, amplifying local neuroinflammation; and it cleaves gasdermin D, whose N‐terminal fragment oligomerizes on the plasma membrane, forming pores that cause osmotic cell lysis and pyroptosis. Given that, Schwann cells are exclusively responsible for myelinating peripheral axons; their pyroptotic death is structurally catastrophic. The loss of viable Schwann cells directly compromises myelin protein synthesis and membrane wrapping, leading to segmental demyelination. Furthermore, the inflammatory milieu generated by pyroptotic Schwann cells enriched with IL‐1*β*, IL‐18, and ATP can sensitize nociceptive nerve endings and recruit additional immune cells, creating a vicious cycle that exacerbates axonal damage. Demyelination, in turn, exposes voltage‐gated potassium channels along the internodes, dissipates the transmembrane ion gradient, and disrupts the saltatory conduction required for rapid action potential propagation, ultimately manifesting as reduced MNCV and thermal hypoesthesia or hyperalgesia [38]. Therefore, the NLRP3 inflammasomes serve as a pivotal link connecting metabolic stress to structural nerve degeneration and functional decline in DPN.

In the context of this mechanistic framework, our molecular data show that EA suppressed the expression of ASC, caspase‐1, and NLRP3, thereby mitigating inflammatory responses and correlating with neuroprotection. Research on acupuncture and NLRP3 inflammasome activation has largely focused on central nervous system disorders, including spinal cord injury [39], cognitive impairment [40], and Alzheimer′s disease [41]. However, given the structural and functional similarities between the central and peripheral nervous systems, recent studies indicate that acupuncture can also protect peripheral nerves and alleviate postherpetic neuralgia via this mechanism [42, 43]. Moreover, the involvement of NLRP3 and caspase‐1 has been demonstrated in acupuncture‐mediated amelioration of diabetes‐induced gastroparesis and dry eye syndrome [44, 45]. These observations further support our conclusion that EA protects peripheral nerves in DPN, with the inhibition of NLRP3 inflammasome being a plausible contributing pathway. Importantly, the suppression of NLRP3 inflammasome components we observed in the EA group was accompanied by preserved myelin ultrastructure and improved nerve conduction, suggesting that the anti‐inflammatory effects of EA may directly translate into structural and functional protection.

Interestingly, although EA did not significantly lower random blood glucose in this study, it still preserved nerve structure and function. Given that hyperglycemia remains one of the primary factors for DPN development as a diabetic complication [46], this finding appears to contradict previous research [47–49]. This discrepancy could be attributed to differences in acupoint selection, EA parameters, and intervention timing. Our protocol primarily targeted ST36 and SP6, which are selected for their effects in relieving pain and protecting peripheral nerves, and may have weaker glucose‐lowering potential than protocols emphasizing metabolic regulation points such as BL20 or Yishu. The 2/15 Hz sparse‐dense wave and the moderate current intensity that induced only slight muscle twitches may preferentially activate endogenous opioid systems for analgesic and anti‐inflammatory effects rather than directly improving insulin sensitivity. Moreover, our short‐term intervention initiated after the establishment of DPN may not have been sufficient to reverse existing glucoregulatory disturbances. However, due to the mixed nature of metabolic disorders and the complexity of peripheral nerve structures, researchers have gradually discovered that hyperglycemia does not necessarily maintain a causal relationship with DPN [50–52]. Therefore, EA may exert direct effects on peripheral nerves, such as local inhibition of NLRP3 inflammasome and attenuation of oxidative stress, independent of systemic glucose control, or possibly through additional metabolic pathways such as lipid profile modulation [53]. Future studies could optimize acupoint combinations and stimulation parameters to achieve both glycemic and neuroprotective benefits. To preliminarily explore the deeper mechanisms underlying EA′s attenuation of DPN, we assessed P62. Research indicates [54] that the autophagy adapter protein P62 specifically recognizes NLRP3 inflammasomes as cargo proteins, binding to them and coentering autophagolysosomes for degradation to suppress inflammatory responses. Our findings revealed significantly reduced P62 levels in the EA group (vs. the model group). Although this reduction is consistent with enhanced autophagic degradation of NLRP3 inflammasomes, it does not alone provide sufficient evidence for increased autophagy. Thus, our current data only suggest a possible involvement of autophagy in the observed effects.

This study still has certain limitations. First, this study lacked a sham EA control group, so nonspecific effects from needling or restraint stress cannot be completely excluded. However, the ST36 and SP6 combination with the EA parameters used here has been shown to exert acupoint‐specific effects in prior DPN studies [28], whereas sham EA controls generally did not yield comparable neuroprotection [55]. This suggests that the observed benefit of EA is primarily attributable to specific acupoint stimulation. Nevertheless, future studies with a rigorous sham EA group are still required to confirm this. Second, we utilized the progression from diabetes to DPN during the DPN modeling process to preliminarily validate that acupuncture has an attenuating effect against DPN development. However, this conclusion remains constrained by the complexity of DPN symptoms and lacks further behavioral and pathological assays to substantiate this statement more definitively. We will employ more diversified and objective assessment methods, such as cold perception testing, mechanical pain testing, and measurement of epidermal nerve fiber density, to refine this conclusion. Third, although we preliminarily hypothesize that autophagy may work in the inhibition of NLRP3 inflammasomes by EA to alleviate inflammatory responses, further validation is lacking. Moving forward, we plan to investigate the regulatory role of EA by incorporating autophagy inhibitors, thereby providing deeper insights into the mechanism by which acupuncture attenuates DPN development. Fourth, this study utilized only male rats, which limits the generalizability of our findings to female subjects, given the well‐documented sex differences in pain perception, inflammatory responses, and neuropathic pain processing. Finally, the TEM analysis of sciatic nerve ultrastructure was performed only qualitatively, without semiquantitative scoring and statistical analysis of demyelination or mitochondrial swelling, which limits the objectivity of the morphological evidence.

Despite these limitations, our study has several notable strengths. The multilevel experimental design integrating behavioral, electrophysiological, ultrastructural, and molecular analyses provides a comprehensive assessment of EA′s neuroprotective effects. The use of a high‐energy diet combined with STZ injection closely mimics human Type 2 DPN. The early initiation of EA intervention after diabetes onset allows evaluation of its potential to attenuate DPN development. The combination of Western blot, RT‐PCR, and ELISA enables simultaneous quantification of protein expression, mRNA levels, and secreted cytokines, offering a multidimensional view of the inflammatory response.

The translational potential of our findings prompts several lines of future inquiry. Causal validation of EA‐mediated NLRP3 inhibition requires NLRP3‐specific inhibitors, activators, or Schwann cell‐targeted genetic models. The role of autophagy should be confirmed using canonical markers ratio and functional assays. Optimization of EA parameters, including acupoint combinations, frequency, intensity, and duration, is needed to identify the most effective protocol. From a clinical standpoint, our findings suggest that EA may serve as an early adjunctive intervention for diabetic patients at risk of DPN. Based on the parameters used here, the acupoint combination of ST36, SP6, and EX‐B3 appears effective for neuroprotection. EA may be particularly suitable for patients with inadequate response or intolerance to pharmacotherapy, given its favorable safety profile. However, clinical application should be individualized, and EA should complement, not replace, standard glycemic control and pharmacological management.

## 5. Conclusion

EA may protect peripheral nerve tissue morphology and function by suppressing NLRP3 inflammasomes, thereby alleviating inflammatory responses and potentially attenuating the onset and progression of DPN. This process may be associated with autophagy, but further mechanistic studies are needed to establish causality.

## Author Contributions

Ziyue Zhu: conceptualization, investigation, and writing—original draft. Qinglan Zou, Weijian Zeng, Nanxi Li, and Bumrungtham Thitinun: formal analysis, validation, visualization. Chengguo Su: methodology, investigation, and resources. Xi You: data curation, software, writing—review and editing. Junpeng Yao and Ying Li: investigation, formal analysis, supervision, writing—review and editing. Wei Zhang: conceptualization, funding acquisition, project administration, supervision, writing—review and editing.

## Funding

This study was supported by Sichuan Provincial Natural Science Foundation Project (2023NSFSC1825) and National Natural Science Foundation of China (10.13039/501100001809; 82474653).

## Ethics Statement

This study was conducted in accordance with an experimental protocol approved by the Animal Welfare and Ethics Committee (2023‐06) of Chengdu University of Traditional Chinese Medicine.

## Conflicts of Interest

The authors declare no conflicts of interest.

## Supporting information


**Supporting Information** Additional supporting information can be found online in the Supporting Information section. File S1 contains Table S1, which summarizes effect sizes and 95% confidence intervals for all nerve function outcomes. Tables S2–S5 present the RT‐PCR data analysis and results. Figures S1–S10 show the amplification curves and melt curves for RT‐PCR. Figures S11–S15 show the Western blot bands. File S2 provides the original data for PWL, MNCV, and blood glucose measurements. All supporting items are cited in the main text.

## Data Availability

The datasets used and/or analyzed during the current study are available from the corresponding authors on reasonable request.

## References

[1] Saeedi P. , Petersohn I. , Salpea P. , Malanda B. , Karuranga S. , Unwin N. , Colagiuri S. , Guariguata L. , Motala A. A. , Ogurtsova K. , Shaw J. E. , Bright D. , Williams R. , and IDF Diabetes Atlas Committee , Global and Regional Diabetes Prevalence Estimates for 2019 and Projections for 2030 and 2045: Results From the International Diabetes Federation Diabetes Atlas, 9(th) edition, Diabetes Research and Clinical Practice. (2019) 157, 107843, 10.1016/j.diabres.2019.107843, 31518657. 31518657

[2] Zhu J. , Hu Z. , Luo Y. , Liu Y. , Luo W. , du X. , Luo Z. , Hu J. , and Peng S. , Diabetic Peripheral Neuropathy: Pathogenetic Mechanisms and Treatment, Frontiers in Endocrinology. (2024) 14, 1265372, 10.3389/fendo.2023.1265372, 38264279. PMC10803883 38264279

[3] Elafros M. A. , Andersen H. , Bennett D. L. , Savelieff M. G. , Viswanathan V. , Callaghan B. C. , and Feldman E. L. , Towards Prevention of Diabetic Peripheral Neuropathy: Clinical Presentation, Pathogenesis, and New Treatments, Lancet Neurology. (2022) 21, no. 10, 922–936, 10.1016/S1474-4422(22)00188-0, 36115364. PMC10112836 36115364

[4] Zochodne D. W. , The Challenges of Diabetic Polyneuropathy: A Brief Update, Current Opinion in Neurology. (2019) 32, no. 5, 666–675, 10.1097/WCO.0000000000000723, 31306212. 31306212

[5] Tavakoli M. , Klingelhöfer D. , Fadavi H. , and Groneberg D. A. , The Landscape of Global Research on Diabetic Neuropathy, Frontiers in Endocrinology. (2023) 14, 1220896, 10.3389/fendo.2023.1220896, 38034004. PMC10686065 38034004

[6] Ismail A. M. A. , Abd Elfatah Abo Saif H. F. , and el-Moatasem Mohamed A. M. , Effect of Jyoti-Trataka on Intraocular Pressure, Autonomic Control, and Blood Glucose in Diabetic Patients With High-Tension primary Open-Angle Glaucoma: A Randomized-Controlled Trial, Journal of Complementary and Integrative Medicine. (2022) 19, no. 4, 1013–1018, 10.1515/jcim-2021-0041, 34303323. 34303323

[7] Elfahl A. M. , Elfeky H. M. , and Ismail A. M. A. , Pilates Versus Gyrotonic Expansion System on Chemotherapy-Induced Peripheral Neuropathy and Fall Risk, EXPLORE. (2026) 22, no. 5, 103475, 10.1016/j.explore.2026.103475, 42425023. 42425023

[8] Han C. , Sun Z. , Yue J. , Zhang Q. , and Wang D. , Systematic Review on the Clinical Efficacy of Acupuncture in the Treatment of Sciatica, Journal of Liaoning University of Traditional Chinese Medicine. (2014) 41, no. 2, 324–326.

[9] Chen Y. , Tong X. , Yang S. , Li W. , and Ma T. , Study on the Expression of Macrophage Migration Inhibitory Factor After Acupuncture Intervention on Peripheral Nerve Injury, China Journal of Traditional Chinese Medicine and Pharmacy. (2020) 35, no. 5, 2577–2581.

[10] Chen Y. , A Study on the Mechanism of Acupuncture in Promoting Nerve Repair in Rats With Sciatic Nerve Injury Based on the JNK Signaling Pathway, 2020, Doctor. Liaoning University of Traditional Chinese Medicine, 10.27213/d.cnki.glnzc.2020.000018.

[11] Huang M. C. , Chang S. C. , Liao W. L. , Ke T. W. , Lee A. L. , Wang H. M. , Chang C. P. , Yen H. R. , Chang H. H. , and Chen W. T. , Acupuncture May Help to Prevent Chemotherapy-Induced Peripheral Neuropathy: A Randomized, Sham-Controlled, Single-Blind Study, Single-Blind Study, Oncologist. (2023) 28, no. 6, e436–e447, 10.1093/oncolo/oyad065, 36971468. PMC10243779 36971468

[12] Hu Q. , Ma Y. , Fei X. , Chen L. H. , Kang Y. R. , Li X. , Chen Z. Y. , Jiang C. L. , Qu S. Y. , Wang H. Z. , and Jiang Y. L. , Effect of Electroacupuncture and Pretreatment of Electroacupuncture on Pain Sensitization and Expression of P2X7R in Spinal Dorsal Horn in Rats With Diabetic Neuropathic Pain, Chinese Acupuncture & Moxibustion. (2022) 42, no. 2, 173–178. 10.13703/j.0255-2930.20210208-k0004 35152582

[13] Baum P. , Toyka K. V. , Blüher M. , Kosacka J. , and Nowicki M. , Inflammatory Mechanisms in the Pathophysiology of Diabetic Peripheral Neuropathy (DN)-New Aspects, International Journal of Molecular Sciences. (2021) 22, no. 19, 10835, 10.3390/ijms221910835, 34639176. PMC8509236 34639176

[14] Stino A. M. , Rumora A. E. , Kim B. , and Feldman E. L. , Evolving Concepts on the Role of Dyslipidemia, Bioenergetics, and Inflammation in the Pathogenesis and Treatment of Diabetic Peripheral Neuropathy, Journal of the Peripheral Nervous System. (2020) 25, no. 2, 76–84, 10.1111/jns.12387, 32412144. PMC7375363 32412144

[15] Xue T. , Zhang X. , Xing Y. , Liu S. , Zhang L. , Wang X. , and Yu M. , Advances About Immunoinflammatory Pathogenesis and Treatment in Diabetic Peripheral Neuropathy, Frontiers in Pharmacology. (2021) 12, 748193, 10.3389/fphar.2021.748193, 34671261. PMC8520901 34671261

[16] Tiwari R. , Wal P. , Singh P. , Tiwari G. , and Rai A. , A Review on Mechanistic and Pharmacological Findings of Diabetic Peripheral Neuropathy including Pharmacotherapy, Current Diabetes Reviews. (2021) 17, no. 3, 247–258, 10.2174/1573399816666200914141558, 32928092. 32928092

[17] Swanson K. V. , Deng M. , and Ting J. P.-Y. , The NLRP3 Inflammasome: Molecular Activation and Regulation to Therapeutics, Nature Reviews Immunology. (2019) 19, no. 8, 477–489, 10.1038/s41577-019-0165-0, 31036962. PMC7807242 31036962

[18] Cheng Y. C. , Chu L. W. , Chen J. Y. , Hsieh S. L. , Chang Y. C. , Dai Z. K. , and Wu B. N. , Loganin Attenuates High Glucose-Induced Schwann Cells Pyroptosis by Inhibiting ROS Generation and NLRP3 Inflammasome Activation, Cells. (2020) 9, no. 9, 10.3390/cells9091948, 32842536. PMC7564733 32842536

[19] Guo H. , Callaway J. B. , and Ting J. P. Y. , Inflammasomes: Mechanism of Action, Role in Disease, and Therapeutics, Nature Medicine. (2015) 21, no. 7, 677–687, 10.1038/nm.3893, 26121197. PMC4519035 26121197

[20] Meade E. and Garvey M. , The Role of Neuro-Immune Interaction in Chronic Pain Conditions; Functional Somatic Syndrome, Neurogenic Inflammation, and Peripheral Neuropathy, International Journal of Molecular Sciences. (2022) 23, no. 15, 10.3390/ijms23158574, 35955708. PMC9369187 35955708

[21] Ismail A. M. A. and El-Azeim A. S. A. , Short-Term Intraocular Pressure Response to the Combined Effect of Transcutaneous Electrical Nerve Stimulation Over Acupoint (Acu-TENS) and Yoga Ocular Exercise in Type 2 Diabetic Patients With Primary Open-Angle Glaucoma: A Randomized Controlled Trial, Innovations in Acupuncture and Medicine. (2021) 14, no. 5, 193–199. 10.51507/j.jams.2021.14.5.193 35770588

[22] Liu S. , Wang Z. , Su Y. , Qi L. , Yang W. , Fu M. , Jing X. , Wang Y. , and Ma Q. , A Neuroanatomical Basis for Electroacupuncture to Drive the Vagal-Adrenal Axis, Nature. (2021) 598, no. 7882, 641–645, 10.1038/s41586-021-04001-4, 34646018. PMC9178665 34646018

[23] Wang L. , Yang J. W. , Lin L. T. , Huang J. , Wang X. R. , Su X. T. , Cao Y. , Fisher M. , and Liu C. Z. , Acupuncture Attenuates Inflammation in Microglia of Vascular Dementia Rats by Inhibiting miR-93-Mediated TLR4/MyD88/NF-*κ*B Signaling Pathway, Oxidative Medicine and Cellular Longevity. (2020) 2020, 15, 10.1155/2020/8253904. PMC7441436 32850002

[24] Ding Y. Y. , Xu F. , Wang Y. F. , Han L. L. , Huang S. Q. , Zhao S. , Ma L. L. , Zhang T. H. , Zhao W. J. , and Chen X. D. , Electroacupuncture Alleviates Postoperative Pain Through Inhibiting Neuroinflammation via Stimulator of Interferon Genes/Type-1 Interferon Pathway, Journal of Integrative Medicine. (2023) 21, no. 5, 496–508, 10.1016/j.joim.2023.07.001, 37517892. 37517892

[25] Zhang J. , Yang X. , Zhang X. , Lu D. , and Guo R. , Electro-Acupuncture Protects Diabetic Nephropathy-Induced Inflammation Through Suppression of NLRP3 Inflammasome in Renal Macrophage Isolation, Endocrine, Metabolic & Immune Disorders Drug Targets. (2021) 21, no. 11, 2075–2083, 10.2174/1871530321666210118161721, 33461476. 33461476

[26] Lamark T. and Johansen T. , Mechanisms of Selective Autophagy, Annual Review of Cell and Developmental Biology. (2021) 37, no. 1, 143–169, 10.1146/annurev-cellbio-120219-035530. 34152791

[27] Pan H. , Wang H. , Wang Y. , and Huang H. , Rules of acupoint Selection for Diabetic Peripheral Neuropathy Based on Data Mining Technology, Chinese Acupuncture & Moxibustion. (2016) 36, no. 10, 1111–1114, 10.13703/j.0255-2930.2016.10.032, 29231536. 29231536

[28] Zhang Z. D. , Wang R. Q. , Liu J. X. , Li X. F. , Zhang X. , Du Y. Z. , Gao F. , Jia C. S. , and Xing H. J. , Characteristics of Clinical Application of Electroacupuncture Therapy for Peripheral Neuropathy Based on Data Mining, Acupuncture Research. (2021) 46, no. 3, 240–247. 10.13702/j.1000-0607.200638 33798299

[29] Yuan C. X. , Wang X. , Zi Y. U. , Lian X. Y. , and Xu B. , Electroacupuncture Improves Peripheral Neuropathy by Up-Regulating Sirt1/PGC-1*α*/TFAM Pathway in Type 2 Diabetes Rats With Peripheral Neuropathy, Acupuncture Research. (2024) 49, no. 4, 349–357. 10.13702/j.1000-0607.20221318 38649202

[30] Zhu Z. , Wang L. , Yao J. , Liu H. , Li Y. , Li Y. , and Zhang W. , Research Progress in Evaluation of Type 2 Diabetic Peripheral Neuropathy Animal Model, Chinese Journal of Comparative Medicine. (2024) 34, no. 1, 139–145.

[31] Liu Y. P. , Yang Y. D. , Mou F. F. , Zhu J. , Li H. , Zhao T. T. , Zhao Y. , Shao S. J. , Cui G. H. , and Guo H. D. , Exosome-Mediated miR-21 Was Involved in the Promotion of Structural and Functional Recovery Effect Produced by Electroacupuncture in Sciatic Nerve Injury, Oxidative Medicine and Cellular Longevity. (2022) 2022, no. 1, 7530102, 10.1155/2022/7530102, 35132352. PMC8817850 35132352

[32] Abdelkader N. F. , Elbaset M. A. , Moustafa P. E. , and Ibrahim S. M. , Empagliflozin Mitigates Type 2 Diabetes-Associated Peripheral Neuropathy: A Glucose-Independent Effect Through AMPK Signaling, Archives of Pharmacal Research. (2022) 45, no. 7, 475–493, 10.1007/s12272-022-01391-5, 35767208. PMC9325846 35767208

[33] Ma X. , Chen Y. , Li X. C. , Mi W. L. , Chu Y. X. , Wang Y. Q. , and Mao-Ying Q. L. , Spinal Neuronal GRK2 Contributes to Preventive Effect by Electroacupuncture on Cisplatin-Induced Peripheral Neuropathy in Mice, Anesthesia and Analgesia. (2022) 134, no. 1, 204–215, 10.1213/ANE.0000000000005768, 34652301. PMC8647702 34652301

[34] Mendonca H. R. , Carpi-Santos R. , da Costa C. K. , and Blanco Martinez A. M. , Neuroinflammation and Oxidative Stress act in Concert to Promote Neurodegeneration in the Diabetic Retina and Optic Nerve: Galectin-3 Participation, Neural Regeneration Research. (2020) 15, no. 4, 625–635, 10.4103/1673-5374.266910, 31638084. PMC6975153 31638084

[35] Fang X. X. , Wang H. , Song H. L. , Wang J. , and Zhang Z. J. , Neuroinflammation Involved in Diabetes-Related Pain and Itch, Frontiers in Pharmacology. (2022) 13, 921612, 10.3389/fphar.2022.921612, 35795572. PMC9251344 35795572

[36] Jayabalan N. , Oronsky B. , Cabrales P. , Reid T. , Caroen S. , Johnson A. M. , Birch N. A. , O’Sullivan J. D. , and Gordon R. , A Review of RRx-001: A Late-Stage Multi-Indication Inhibitor of NLRP3 Activation and Chronic Inflammation, Drugs. (2023) 83, no. 5, 389–402, 10.1007/s40265-023-01838-z, 36920652. PMC10015535 36920652

[37] Péladeau C. and Sandhu J. K. , Aberrant NLRP3 Inflammasome Activation Ignites the Fire of Inflammation in Neuromuscular Diseases, International Journal of Molecular Sciences. (2021) 22, no. 11, 10.3390/ijms22116068, 34199845. PMC8200055 34199845

[38] Feng H. , Ding R. , Zhou Q. , Wu T. C. , Li H. , Ma X. T. , and Yue R. S. , Role of NLRP3 Inflammasome in Diabetic Neuropathy and Prevention and Treatment With Traditional Chinese Medicine, China Journal of Chinese Materia Medica. (2023) 48, no. 2, 300–310. 10.19540/j.cnki.cjcmm.20220905.707 36725219

[39] Chen Y. , Wu L. , Shi M. , Zeng D. , Hu R. , Wu X. , Han S. , He K. , Xu H. , Shao X. M. , and Ma R. , Electroacupuncture Inhibits NLRP3 Activation by Regulating CMPK2 After Spinal Cord Injury, Frontiers in Immunology. (2022) 13, 788556, 10.3389/fimmu.2022.788556, 35401582. PMC8987202 35401582

[40] Sun L. , Yong Y. , Wei P. , Wang Y. , Li H. , Zhou Y. , Ruan W. , Li X. , and Song J. , Electroacupuncture Ameliorates Postoperative Cognitive Dysfunction and Associated Neuroinflammation via NLRP3 Signal Inhibition in Aged Mice, CNS Neuroscience & Therapeutics. (2022) 28, no. 3, 390–400, 10.1111/cns.13784, 34951130. PMC8841296 34951130

[41] Zhu H. , Zhang T. , Li R. , Ren D. , Xu J. , and Xiao L. , Bushen Huoxue Acupuncture Ameliorates Alzheimer′s Disease by Upregulating MARCHF3 to Induce NLRP3 Ubiquitination and Inhibit Caspase-1-Dependent Pyroptosis, Metabolic Brain Disease. (2025) 40, no. 1, 10.1007/s11011-024-01459-9, 39556273. PMC11573812 39556273

[42] Zhang H. , Xiang H. C. , Jia M. , Lei C. , Hou T. F. , and Chen B. G. , Mechanism on the Involvement of CB2 Receptor in Inhibiting the Activation of NLRP3 Inflammasome by Electroacupuncture on Alleviating Neuropathic Pain After Paclitaxel Chemotherapy, China Journal of Traditional Chinese Medicine and Pharmacy. (2018) 33, no. 5, 2103–2107.

[43] Qiu Y. , Xie F. , Gao C. , and JiaminHuang T. T. , The Effect of Warm Acupuncture on the NLRP3/Caspase1 Pathway and Related Inflammatory Factors in Rats With Post-Herpetic Neuralgia due to Kidney Yang Deficiency, Journal of Guizhaou University of Traditional Chinese Medicine. (2024) 46, no. 6, 34–42.

[44] Xiao L. , Wei X. , Zhang T. , Li X. Y. , Yu X. M. , and Zhao S. T. , Effect of Electroacupuncture at Zusanli (ST 36), Liangmen (ST 21) and Sanyinjiao (SP 6) on NLRP3 Inflammasome and Downstream Inflammatory Factors in Diabetic Gastroparesis Rats, Journal of Traditional Chinese Medicine. (2023) 64, no. 9, 948–953.

[45] Wan M. , Fu Z. , He L. , Sun X. , and Gao W. , The Regulatory Role of Electroacupuncture on Caspase-1 and Its Downstream IL-1*β* and IL-18 in Cornea of Type 2 Diabetic Dry Eye Rats, World Science and Technology-Modernization of Traditional Chinese Medicine. (2024) 26, no. 11, 2905–2913.

[46] Wang W. , Ji Q. , Ran X. , Li C. , Kuang H. , Yu X. , Fang H. , Yang J. , Liu J. , Xue Y. , Feng B. , Lei M. , and Zhu D. , Prevalence and Risk Factors of Diabetic Peripheral Neuropathy: A Population-Based Cross-Sectional Study in China, Diabetes/Metabolism Research and Reviews. (2023) 39, no. 8, e3702, 10.1002/dmrr.3702, 37490047. 37490047

[47] Cao B. , Li R. , Tian H. , Jia N. , Hu X. G. , Ma Y. J. , and Wang Y. Y. , Effect of Electroacupuncture at "Weiwanxiashu" (EX-B 3) on Islet Morphology and the Expression of Pancreatic Glucagon-Like Peptide-1 Receptor in Type 2 Diabetes Rats, Acupuncture Research. (2017) 42, no. 2, 107–113. 29071956

[48] Cao B. , Li R. , Tian H. , Hu X. , Jia N. , Ma Y. , and Wang Y. , Experimental Study of Glucose-Lowering Effects on Electroacupuncture at Back-Shu Points in Rats, Chinese Acupuncture & Moxibustion. (2016) 36, no. 12, 1283–1287, 10.13703/j.0255-2930.2016.12.016, 29231367. 29231367

[49] Ali isMAil A. M. and Abd El-Azeim A. S. , Immediate Fasting Blood Glucose Response to Electroacupuncture of ST36 Versus CV12 in Patients With Type 2 Diabetes Mellitus: Randomized Controlled Trial, Family Medicine & Primary Care Review. (2021) 23, no. 4, 437–441, 10.5114/fmpcr.2022.110370.

[50] Martin C. L. , Albers J. W. , Pop-Busui R. , and for the DCCT/EDIC Research Group , Neuropathy and Related Findings in the Diabetes Control and Complications Trial/Epidemiology of Diabetes Interventions and Complications Study, Diabetes Care. (2014) 37, no. 1, 31–38, 10.2337/dc13-2114, 24356595. PMC3868000 24356595

[51] Wang L. , Chopp M. , Jia L. , Lu X. , Szalad A. , Zhang Y. , Zhang R. L. , and Zhang Z. G. , Therapeutic Benefit of Extended Thymosin*β*4 Treatment Is Independent of Blood Glucose Level in Mice with Diabetic Peripheral Neuropathy, Journal Diabetes Research. (2015) 2015, 173656, 10.1155/2015/173656. PMC4405294 25945352

[52] Calcutt N. A. , Diabetic Neuropathy and Neuropathic Pain: A (con)Fusion of Pathogenic Mechanisms?, Pain. (2020) 161, no. Supplement 1, S65–s86, 10.1097/j.pain.0000000000001922, 32999525. PMC7521457 32999525

[53] Ali Ismail A. M. , Lipid Profile Response to Acupuncture in Obese Patients With Subjective Tinnitus: A Randomized Controlled Trial, Journal of Acupuncture and Meridian Studies. (2023) 16, no. 1, 11–19, 10.51507/j.jams.2023.16.1.11, 36804817. 36804817

[54] Mehto S. , Jena K. K. , Nath P. , Chauhan S. , Kolapalli S. P. , das S. K. , Sahoo P. K. , Jain A. , Taylor G. A. , and Chauhan S. , The Crohn′s Disease Risk Factor IRGM Limits NLRP3 Inflammasome Activation by Impeding Its Assembly and by Mediating Its Selective Autophagy, Molecular Cell. (2019) 73, no. 3, 429–445, 10.1016/j.molcel.2018.11.018, 30612879. PMC6372082 30612879

[55] Pérez Hernández M. F. , Rodríguez Guerrero E. , Ocharan Hernández M. E. , Gómez Jiménez D. C. , Gómez Esquivel M. L. , Aguilar Castillo S. J. , Flores Hernández V. D. , Romo Dueñas S. , Torres Mendoza M. R. , Calderón Vallejo A. , Velázquez García G. , Moreno Tovar M. G. , Cruz López M. , Ordóñez Rodríguez J. M. , Peña Juárez K. A. , Miranda Gastelum X. , Castro Díaz J. M. , Aguirre Guerrero A. , Macías Zaragoza V. M. , and Peralta Romero J. J. , Effect of Electroacupuncture for Pain Relief in Diabetic Distal Symmetric Polyneuropathy: A Pilot Randomized Clinical Trial, BMC Complementary Medicine and Therapies. (2025) 25, no. 1, 10.1186/s12906-025-05135-y, 41366387. PMC12690971 41366387

