# Supplementary material for: Electroacupuncture Attenuates Peripheral Neuropathy Development in Hyperglycemic Rats and Is Associated With NLRP3 Inflammasome Modulation
Source: J Diabetes Res. 2026 Oct 19;2026:6233881. doi: 10.1155/jdr/6233881 (PMC13678854; doi:10.1155/jdr/6233881)
Supplement: Supplementary file 1 [file JDR-2026-6233881-s001.zip › 6233881.f1/Supplementary File 1.docx]

### **Supplementary Table S1. Effect sizes and pairwise comparisons for all outcomes.**

| Outcome | Partial η² | Comparison | Mean difference | 95% CI | P value |
| --- | --- | --- | --- | --- | --- |
| **Blood glucose** | 0.94 | Model vs. Control | 25.04 | [21.16, 28.92] | <0.0001 |
|  |  | EA vs. Control | 24.57 | [20.69, 28.45] | <0.0001 |
|  |  | EA vs. Model | 0.47 | [–3.41, 4.35] | 0.95 |
| **PWL** | 0.64 | Model vs. Control | 7.75 | [4.32, 11.18] | <0.0001 |
|  |  | EA vs. Control | 1.45 | [–1.98, 4.88] | 0.59 |
|  |  | EA vs. Model | –6.30 | [–9.73, –2.87] | <0.0001 |
| **MNCV** | 0.82 | Model vs. Control | –13.27 | [–18.50, –8.04] | <0.0001 |
|  |  | EA vs. Control | –2.47 | [–7.70, 2.76] | >0.05 |
|  |  | EA vs. Model | 10.80 | [5.57, 16.03] | <0.0001 |
| **ASC protein** | 0.73 | Model vs. Control | 3.05 | [1.38, 4.72] | <0.01 |
|  |  | EA vs. Control | 0.00 | [–1.67, 1.67] | >0.05 |
|  |  | EA vs. Model | –3.05 | [–4.72, –1.38] | <0.01 |
| **Caspase-1 protein** | 0.76 | Model vs. Control | 3.68 | [1.99, 5.37] | <0.0001 |
|  |  | EA vs. Control | 0.75 | [–0.94, 2.44] | >0.05 |
|  |  | EA vs. Model | –2.93 | [–4.62, –1.24] | <0.0001 |
| **NLRP3 protein** | 0.65 | Model vs. Control | 2.69 | [1.02, 4.36] | <0.0001 |
|  |  | EA vs. Control | 0.38 | [–1.29, 2.05] | >0.05 |
|  |  | EA vs. Model | –2.31 | [–3.98, –0.64] | <0.001 |
| **P62 protein** | 0.79 | Model vs. Control | 2.02 | [1.17, 2.87] | <0.001 |
|  |  | EA vs. Control | 0.43 | [–0.42, 1.28] | >0.05 |
|  |  | EA vs. Model | –1.59 | [–2.44, –0.74] | <0.0001 |
| **ASC mRNA** | 0.71 | Model vs. Control | 0.58 | [0.29, 0.87] | <0.001 |
|  |  | EA vs. Control | 0.19 | [–0.10, 0.48] | >0.05 |
|  |  | EA vs. Model | –0.39 | [–0.68, –0.10] | <0.05 |
| **Caspase-1 mRNA** | 0.44 | Model vs. Control | 1.01 | [0.11, 1.91] | <0.01 |
|  |  | EA vs. Control | 0.33 | [–0.57, 1.23] | >0.05 |
|  |  | EA vs. Model | –0.68 | [–1.58, 0.22] | <0.05 |
| **NLRP3 mRNA** | 0.31 | Model vs. Control | 0.63 | [–0.18, 1.44] | <0.05 |
|  |  | EA vs. Control | 0.04 | [–0.77, 0.85] | >0.05 |
|  |  | EA vs. Model | –0.59 | [–1.40, 0.22] | <0.05 |
| **P62 mRNA** | 0.51 | Model vs. Control | 0.67 | [0.15, 1.19] | <0.001 |
|  |  | EA vs. Control | 0.25 | [–0.27, 0.77] | >0.05 |
|  |  | EA vs. Model | –0.42 | [–0.94, 0.10] | <0.05 |
| **IL-18** | 0.65 | Model vs. Control | 19.62 | [8.05, 31.19] | <0.001 |
|  |  | EA vs. Control | 5.64 | [–5.93, 17.21] | >0.05 |
|  |  | EA vs. Model | –13.98 | [–25.55, –2.41] | <0.01 |
| **IL-1β** | 0.75 | Model vs. Control | 108.20 | [56.09, 160.31] | <0.0001 |
|  |  | EA vs. Control | 18.70 | [–33.41, 70.81] | >0.05 |
|  |  | EA vs. Model | –89.50 | [–141.61, –37.39] | <0.0001 |

## RT-PCR data analysis and results

**Table S2. Analysis of ASC relative expression**

|  | **Gene name** | | **△CT=CT_target gene_-Ct_reference gene_** | **△CT_Control_** | **△△CT=△CT_treatment_-△CT_Control_** | **Fold change=2^-△△CT^(Normalized expression)** |
| --- | --- | --- | --- | --- | --- | --- |
| Sample ID | β-actin | ASC |  |  |  |  |
| Control-11 | 19.596 | 28.550 | 8.95 |  | 0.16 | 0.90 |
| Control-26 | 18.772 | 27.825 | 9.05 |  | 0.26 | 0.84 |
| Control-9 | 19.424 | 28.196 | 8.77 |  | -0.03 | 1.02 |
| Control-10 | 19.555 | 28.233 | 8.68 |  | -0.12 | 1.09 |
| Control-7 | 20.233 | 28.942 | 8.71 |  | -0.09 | 1.06 |
| Control-8 | 19.875 | 28.249 | 8.37 |  | -0.42 | 1.34 |
| Control-2 | 18.763 | 27.613 | 8.85 |  | 0.05 | 0.96 |
| Control-4 | 18.992 | 27.977 | 8.98 | 8.80 | 0.19 | 0.88 |
| Model-75 | 20.356 | 28.744 | 8.39 |  | -0.41 | 1.33 |
| Model-25 | 21.189 | 29.124 | 7.94 |  | -0.86 | 1.82 |
| Model-29 | 20.371 | 28.446 | 8.08 |  | -0.72 | 1.65 |
| Model-32 | 21.624 | 29.576 | 7.95 |  | -0.85 | 1.80 |
| Model-81 | 19.683 | 27.852 | 8.17 |  | -0.63 | 1.55 |
| Model-74 | 19.179 | 27.491 | 8.31 |  | -0.49 | 1.40 |
| Model-12 | 21.491 | 29.548 | 8.06 |  | -0.74 | 1.67 |
| Model-76 | 19.768 | 27.951 | 8.18 |  | -0.61 | 1.53 |
| EA-24 | 18.341 | 27.348 | 9.01 |  | 0.21 | 0.86 |
| EA-31 | 19.505 | 28.040 | 8.53 |  | -0.26 | 1.20 |
| EA-27 | 21.400 | 29.832 | 8.43 |  | -0.37 | 1.29 |
| EA-23 | 19.557 | 28.159 | 8.60 |  | -0.20 | 1.14 |
| EA-33 | 20.818 | 29.328 | 8.51 |  | -0.29 | 1.22 |
| EA-16 | 20.172 | 28.650 | 8.48 |  | -0.32 | 1.25 |
| EA-17 | 20.432 | 28.804 | 8.37 |  | -0.42 | 1.34 |
| EA-19 | 20.575 | 29.013 | 8.44 |  | -0.36 | 1.28 |

**Table S3. Analysis of Caspase-1 relative expression**

|  | **Gene name** | | **△CT=CT_target gene_-Ct_reference gene_** | **△CT_Control_** | **△△CT=△CT_treatment_-△CT_Control_** | **Fold change=2^-△△CT^(Normalized expression)** |
| --- | --- | --- | --- | --- | --- | --- |
| Sample ID | β-actin | caspase-1 |  |  |  |  |
| Control-11 | 19.596 | 32.446 | 12.85 |  | 0.12 | 0.92 |
| Control-26 | 18.772 | 30.731 | 11.96 |  | -0.77 | 1.71 |
| Control-9 | 19.424 | 32.030 | 12.61 |  | -0.12 | 1.09 |
| Control-10 | 19.555 | 32.297 | 12.74 |  | 0.01 | 0.99 |
| Control-7 | 20.233 | 33.860 | 13.63 |  | 0.90 | 0.54 |
| Control-8 | 19.875 | 32.716 | 12.84 |  | 0.11 | 0.93 |
| Control-2 | 18.763 | 30.616 | 11.85 |  | -0.88 | 1.84 |
| Control-4 | 18.992 | 32.346 | 13.35 | 12.73 | 0.62 | 0.65 |
| Model-75 | 20.356 | 32.323 | 11.97 |  | -0.76 | 1.70 |
| Model-25 | 21.189 | 33.343 | 12.15 |  | -0.58 | 1.49 |
| Model-29 | 20.371 | 31.736 | 11.36 |  | -1.36 | 2.57 |
| Model-32 | 21.624 | 33.960 | 12.34 |  | -0.39 | 1.31 |
| Model-81 | 19.683 | 30.775 | 11.09 |  | -1.64 | 3.11 |
| Model-74 | 19.179 | 30.573 | 11.39 |  | -1.34 | 2.52 |
| Model-12 | 21.491 | 32.884 | 11.39 |  | -1.34 | 2.53 |
| Model-76 | 19.768 | 31.909 | 12.14 |  | -0.59 | 1.50 |
| EA-24 | 18.341 | 30.114 | 11.77 |  | -0.96 | 1.94 |
| EA-31 | 19.505 | 31.374 | 11.87 |  | -0.86 | 1.82 |
| EA-27 | 21.400 | 34.082 | 12.68 |  | -0.05 | 1.03 |
| EA-23 | 19.557 | 30.927 | 11.37 |  | -1.36 | 2.57 |
| EA-33 | 20.818 | 33.739 | 12.92 |  | 0.19 | 0.88 |
| EA-16 | 20.172 | 32.691 | 12.52 |  | -0.21 | 1.16 |
| EA-17 | 20.432 | 32.866 | 12.43 |  | -0.29 | 1.23 |
| EA-19 | 20.575 | 32.956 | 12.38 |  | -0.35 | 1.27 |

**Table S4. Analysis of NLRP3 relative expression**

|  | **Gene name** | | **△CT=CT_target gene_-Ct_reference gene_** | **△CT_Control_** | **△△CT=△CT_treatment_-△CT_Control_** | **Fold change=2^-△△CT^(Normalized expression)** |
| --- | --- | --- | --- | --- | --- | --- |
| Sample ID | β-actin | NLRP3 |  |  |  |  |
| Control-11 | 19.596 | 30.263 | 10.67 |  | 0.46 | 0.72 |
| Control-26 | 18.772 | 28.596 | 9.82 |  | -0.38 | 1.30 |
| Control-9 | 19.424 | 30.389 | 10.96 |  | 0.76 | 0.59 |
| Control-10 | 19.555 | 29.844 | 10.29 |  | 0.09 | 0.94 |
| Control-7 | 20.233 | 31.021 | 10.79 |  | 0.58 | 0.67 |
| Control-8 | 19.875 | 29.722 | 9.85 |  | -0.36 | 1.28 |
| Control-2 | 18.763 | 28.015 | 9.25 |  | -0.95 | 1.93 |
| Control-4 | 18.992 | 28.987 | 9.99 | 10.20 | -0.21 | 1.16 |
| Model-75 | 20.356 | 29.991 | 9.63 |  | -0.57 | 1.48 |
| Model-25 | 21.189 | 31.137 | 9.95 |  | -0.26 | 1.19 |
| Model-29 | 20.371 | 30.215 | 9.84 |  | -0.36 | 1.28 |
| Model-32 | 21.624 | 31.411 | 9.79 |  | -0.42 | 1.34 |
| Model-81 | 19.683 | 28.739 | 9.06 |  | -1.15 | 2.22 |
| Model-74 | 19.179 | 28.413 | 9.23 |  | -0.97 | 1.96 |
| Model-12 | 21.491 | 31.278 | 9.79 |  | -0.42 | 1.34 |
| Model-76 | 19.768 | 28.807 | 9.04 |  | -1.17 | 2.24 |
| EA-24 | 18.341 | 27.682 | 9.34 |  | -0.86 | 1.82 |
| EA-31 | 19.505 | 29.183 | 9.68 |  | -0.53 | 1.44 |
| EA-27 | 21.400 | 31.764 | 10.36 |  | 0.16 | 0.89 |
| EA-23 | 19.557 | 29.246 | 9.69 |  | -0.51 | 1.43 |
| EA-33 | 20.818 | 31.212 | 10.39 |  | 0.19 | 0.88 |
| EA-16 | 20.172 | 30.774 | 10.60 |  | 0.40 | 0.76 |
| EA-17 | 20.432 | 30.306 | 9.87 |  | -0.33 | 1.26 |
| EA-19 | 20.575 | 31.075 | 10.50 |  | 0.30 | 0.81 |

**Table S5. Analysis of P62 relative expression**

|  | **Gene name** | | **△CT=CT_target gene_-Ct_reference gene_** | **△CT_Control_** | **△△CT=△CT_treatment_-△CT_Control_** | **Fold change=2^-△△CT^(Normalized expression)** |
| --- | --- | --- | --- | --- | --- | --- |
| Sample ID | β-actin | P62 |  |  |  |  |
| Control-11 | 19.596 | 25.355 | 5.76 |  | -0.01 | 1.01 |
| Control-26 | 18.772 | 24.660 | 5.89 |  | 0.12 | 0.92 |
| Control-9 | 19.424 | 25.199 | 5.77 |  | 0.00 | 1.00 |
| Control-10 | 19.555 | 25.737 | 6.18 |  | 0.41 | 0.75 |
| Control-7 | 20.233 | 25.251 | 5.02 |  | -0.75 | 1.68 |
| Control-8 | 19.875 | 25.698 | 5.82 |  | 0.05 | 0.96 |
| Control-2 | 18.763 | 24.607 | 5.84 |  | 0.07 | 0.95 |
| Control-4 | 18.992 | 24.864 | 5.87 | 5.77 | 0.10 | 0.93 |
| Model-75 | 20.356 | 25.420 | 5.06 |  | -0.71 | 1.63 |
| Model-25 | 21.189 | 25.905 | 4.72 |  | -1.05 | 2.08 |
| Model-29 | 20.371 | 25.871 | 5.50 |  | -0.27 | 1.21 |
| Model-32 | 21.624 | 26.547 | 4.92 |  | -0.85 | 1.80 |
| Model-81 | 19.683 | 25.005 | 5.32 |  | -0.45 | 1.36 |
| Model-74 | 19.179 | 24.451 | 5.27 |  | -0.50 | 1.41 |
| Model-12 | 21.491 | 26.196 | 4.70 |  | -1.07 | 2.09 |
| Model-76 | 19.768 | 25.015 | 5.25 |  | -0.52 | 1.44 |
| EA-24 | 18.341 | 24.158 | 5.82 |  | 0.05 | 0.97 |
| EA-31 | 19.505 | 24.926 | 5.42 |  | -0.35 | 1.27 |
| EA-27 | 21.400 | 26.389 | 4.99 |  | -0.78 | 1.72 |
| EA-23 | 19.557 | 25.591 | 6.03 |  | 0.26 | 0.83 |
| EA-33 | 20.818 | 25.810 | 4.99 |  | -0.78 | 1.72 |
| EA-16 | 20.172 | 26.271 | 6.10 |  | 0.33 | 0.80 |
| EA-17 | 20.432 | 25.952 | 5.52 |  | -0.25 | 1.19 |
| EA-19 | 20.575 | 26.048 | 5.47 |  | -0.30 | 1.23 |

**Amplification curve and Melt curve**

|  |  |
| --- | --- |
| **Figure S1. Amplification curve of β-actin** | **Figure S6. Melt curve of β-actin** |
|  |  |
| **Figure S2. Amplification curve of ASC** | **Figure S7. Melt curve of ASC** |
|  |  |
| **Figure S3. Amplification curve of Caspase-1** | **Figure S8. Melt curve of Caspase-1** |
|  |  |
| **Figure S4. Amplification curve of NLRP3** | **Figure S9. Melt curve of NLRP3** |
|  |  |
| **Figure S5. Amplification curve of P62** | **Figure S10. Melt curve of P62** |

**Western blot bands**

C2 M12 EA17

C11 M25 EA19

C4 M29	EA23

C26 M32 EA24

C7 M74 EA27

C8 M75 EA31

C9 M76 EA33

C10 M81 EA80

**Supplementary Figure S11. Representative Western blots of β-actin(~41 kDa)**

C2 M12 EA17

C11 M25 EA19

C4 M29	EA23

C26 M32 EA24

C7 M74 EA27

C8 M75 EA31

C9 M76 EA33

C10 M81 EA80

**Supplementary Figure S12. Representative Western blots of ASC(25-33 kDa)**

C2 M12 EA17

C11 M25 EA19

C4 M29	EA23

C26 M32 EA24

C7 M74 EA27

C8 M75 EA31

C9 M76 EA33

C10 M81 EA80

**Supplementary Figure S13. Representative Western blots of Caspase-1 (41-52 kDa)**

C2 M12 EA17

C11 M25 EA19

C4 M29	EA23

C26 M32 EA24

C7 M74 EA27

C8 M75 EA31

C9 M76 EA33

C10 M81 EA80

**Supplementary Figure S14. Representative Western blots of NLRP3 (~140 kDa)**

3.5 P62 (52-65 kDa)

C2 M12 EA17

C11 M25 EA19

C4 M29	EA23

C26 M32 EA24

C7 M74 EA27

C8 M75 EA31

C9 M76 EA33

C10 M81 EA80

**Supplementary Figure S15. Representative Western blots of P62 (52-65 kDa)**
